# Antileishmanial Activity of Amphotericin B-loaded-PLGA Nanoparticles: An Overview

**DOI:** 10.3390/ma11071167

**Published:** 2018-07-09

**Authors:** Ernesto Palma, Antonella Pasqua, Agnese Gagliardi, Domenico Britti, Massimo Fresta, Donato Cosco

**Affiliations:** 1 Department of Health Sciences, University “Magna Græcia” of Catanzaro, Campus Universitario “S. Venuta”, Viale S. Venuta, I-88100 Catanzaro, Italy; palma@unicz.it (E.P.); antonellapasqua@unicz.it (A.P.); britti@unicz.it (D.B.); 2 Department of Experimental and Clinical Medicine, University “Magna Græcia” of Catanzaro, Campus Universitario “S. Venuta”, Viale S. Venuta, I-88100 Catanzaro, Italy; gagliardi@unicz.it

**Keywords:** *Leishmania*, amphotericin B, nanoparticles, PLGA, colloids, drug delivery

## Abstract

In recent decades, nanotechnology has made phenomenal strides in the pharmaceutical field, favouring the improvement of the biopharmaceutical properties of many active compounds. Many liposome-based formulations containing antitumor, antioxidant and antifungal compounds are presently on the market and are used daily (for example Doxil^®^/Caelyx^®^ and Ambisome^®^). Polymeric nanoparticles have also been used to entrap many active compounds with the aim of improving their pharmacological activity, bioavailability and plasmatic half-life while decreasing their side effects. The modulation of the structural/morphological properties of nanoparticles allows us to influence various technological parameters, such as the loading capacity and/or the release profile of the encapsulated drug(s). Amongst the biocompatible polymers, poly(D,L-lactide) (PLA), poly(D,L-glycolide) (PLG) and their co-polymers poly(lactide-co-glycolide) (PLGA) are the most frequently employed due to their approval by the FDA for human use. The aim of this review is to provide a description of the foremost recent investigations based on the encapsulation of amphotericin B in PLGA nanoparticles, in order to furnish an overview of the technological properties of novel colloidal formulations useful in the treatment of Leishmaniasis. The pharmacological efficacy of the drug after nanoencapsulation will be compared to the commercial formulations of the drug (i.e., Fungizone^®^, Ambisome^®^, Amphocil^®^ and Abelcet^®^).

## 1. Introduction

Several species of protozoan parasites (*Leishmania* spp.) are responsible for a wide spectrum of zoonotic diseases called leishmaniasis. A total of 98 countries and three territories on five continents have reported endemic leishmaniasis transmission with ensuing human health threats; in fact, 12 million people are already infected and 350 million people are at risk at the moment, while ~7000 deaths are annually attributed to this pathology [1,2]. Dogs are considered to be the main reservoir of the responsible parasites but other wild and domestic hosts, such as rodents (zoonotic leishmaniasis) or even humans (anthroponotic leishmaniasis) can favor the diffusion of the pathology [3].

**Figure 1:**
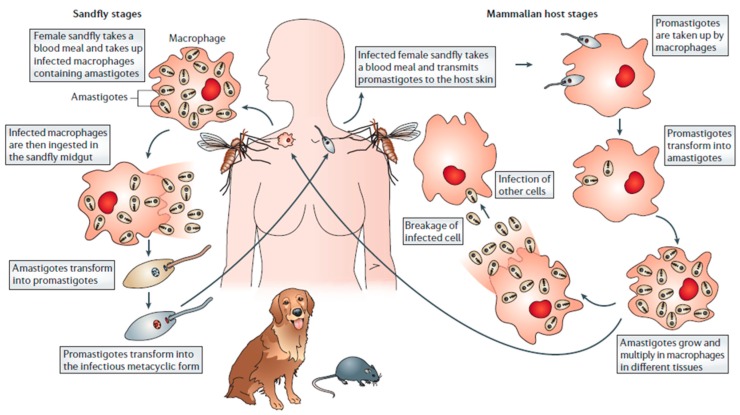
Schematic representation of the infection cycle of leishmaniasis (from [5]).

Leishmaniasis has traditionally been classified into three clinical forms, which are: visceral (VL), cutaneous (CL), and mucocutaneous leishmaniasis (MCL). These differ in immunopathology, degree of morbidity and mortality [4]. As shown in Figure 1, all types of leishmaniasis start with the bite of infected female sandflies, which inject metacyclic promastigotes into the skin of the vertebrate host. These promastigotes penetrate into the macrophages, where they transform into replicating amastigotes. The infected macrophages are taken up by sandflies, where the parasites mature into the infective metacyclic promastigote form [5].

The advent of pentavalent antimonials was a milestone in antileishmanial chemotherapy, especially in endemic countries such as the Indian subcontinent [6]. Branded sodium stibogluconate and meglumine antimoniate are efficacious alternatives of generic antimonials. Although pentavalent antimonials are still the first-line drugs against all forms of leishmaniasis, their clinical use has several limitations. Antimony-based therapy is often characterized by local pain during intramuscular injection followed by systemic side effects, which require very careful medical supervision. Typical side-effects include nausea, vomiting, weakness and myalgia, abdominal colic, diarrhea, skin rashes, and hepatic- and cardio-toxicity [7]. The increasing incidence of resistant strains requires novel formulations for the use of these drugs in endemic areas [8,9,10]. Moreover, the use of these compounds does not provide significant therapeutic effects in infected dogs either, and is unable to prevent frequent relapses, besides necessitating repeated drug administrations, which are both poorly tolerated and expensive [11].

The most important antimonial-based compounds presently in clinical use are complexes of Sb^V^ with *N*-methyl-d-glucamine (meglumine antimoniate or Glucantime^®^) or sodium gluconate (sodium stibogluconate or Pentostam^®^). The mechanism of action of the pentavalent antimonials against leishmaniasis still remains unclear, nor is it clear whether the final active form of antimonials is Sb^(V)^ or Sb^(III)^ [12]. Two models of action have been theorized: (i) that the “Prodrug model”, Sb^(V)^ would act as a prodrug, being reduced to the more toxic Sb^(III)^; and (ii) the “Active Sb^(V)^ model”, that is, Sb^(V)^, which exhibits intrinsic antileishmanial activity [7].

Second-line drugs include amphotericin B (AmB), the drug of choice in the areas with high antimonial resistance [13]. Despite its high degree of efficiency, AmB is also associated with toxicity and the emergence of parasitic resistance [14]. Other drugs such as paromomycin and pentamidine provide evidence of good antifungal activity, and are valid candidates for unconventional and adjuvant therapy even though their use and availability in *Leishmania* endemic regions is limited [15].

Miltefosine, an alkyl-phosphocholine analogue originally developed as a neoplastic agent, was the first drug to be used for the oral treatment of leishmaniasis and was considered the most promising formulation in antileishmanial chemotherapy [16]. Although it does have good pharmacological efficacy, it is very expensive and is characterized by a long half-life that favors the appearance of resistance phenomena and teratogenic side effects [17].

Domperidone, a dopamine D2 receptor antagonist, has been included in the list of anti-*Leishmania* drugs in the current consensus guidelines for the treatment of canine leishmaniasis [18]. Repeated administration of this drug can trigger the cell-mediated immune response in treated dogs through an increase of prolactin in the blood [19,20].

Allopurinol is another compound widely used in the treatment of canine leishmaniasis, both alone (monotherapy) and in association with pentavalent antimonials as a consequence of its relative non-toxicity, its low cost and the fact that it can be administered orally. Despite improved rates of clinical remission, combination antimonial/allopurinol therapy does not avoid recidivation phenomena, but it may temporarily alter the potential infectivity of dogs as regards vectors [21].

Thus, it is in this context that the need to develop novel antileishmanial drugs and/or vaccines is recognized, in order to achieve innovative medicines with the aim of improving the therapeutic responses of pharmacologically efficacious candidates.

### 1.1. AmB Pharmaceutical Formulations

AmB is a polyene antibiotic and it is believed to be leishmanicidal due to its capacity to bind ergosterol, which is the most important sterol in *Leishmania*. The antileishmanial activity of AmB is due to its interaction with the ergosterol of *Leishmania* and the cholesterol of host macrophages. The interaction of AmB with ergosterol leads to the formation of transmembranic AmB channels, which alter its permeability to cations, water and glucose, besides affecting membrane-bound enzymes [22]. Despite its great efficiency, AmB is poorly tolerated. Its administration is limited by infusion-related toxicity, an effect probably resulting from pro-inflammatory cytokine production by innate immune cells. The main acute side effects of AmB are nausea, vomiting, rigors, fever, hypertension/hypotension, and hypoxia [14]. AmB is nephrotoxic and its damaging effect on renal tubular cells is mainly due to several factors, including increased salt and Ca^2+^ concentration and H^+^ permeability across the aqueous pores, which leads to sustained collapse of the pH and Ca^2+^ gradients across the membrane, a mechanism responsible for apoptosis in eukaryotic cells [4].

Novel AmB formulations were realized and marketed in the 1990s in order to decrease the drug’s toxicity and increase its efficacy. Specifically, through the use of specific surfactants, and lipidic and colloidal carriers, several AmB-based nanomedicines (i.e., Fungizone^®^, AmBisome^®^, Abelcet^®^ and Amphotec^®^, see Table 1) were obtained, which were characterized by a suitable efficacy/toxicity ratio [23].

Fungizone^®^ is considered to be the “gold standard” formulation of AmB. It is a hydrophilic colloidal dispersion made up of an anionic surfactant, sodium deoxycholate (SD) (AmB/SD ratio 1:2), organized in micelles suitable for parenteral administration. Fungizone^®^ is a broad-spectrum pharmaceutical, useful for the treatment of fungal infections, but it has provided evidence of several side effects, including renal disorders. It has been suggested that prolonging the infusion time from the usual 4 up to 24 h [23] might be helpful in reducing these side effects. The mixture of AmB and commercial lipid emulsions, administered parenterally as a dietary supplement (e.g., Lipofundin^®^, Intralipid^®^), modified the AmB distribution in vivo [24]. It has been demonstrated that, in AIDS patients with candidiasis, Fungizone^®^ infused with Intralipid^®^ is better tolerated and is less nephrotoxic than the formulation diluted in 5% (*w*/*v*) glucose [25].

Abelcet^®^ is a lipid complex made up of two phospholipids (L-α-dimyristoylphosphatidylcholine and L-α-dimyristoylphosphatidylglycerol) and AmB. The therapeutic index of Abelcet^®^ is better than that of Fungizone^®^, but the lipid complex is quickly removed from the blood stream by the cells of the mononuclear phagocyte system (MPS), thus increasing the risk of hepatic disorders while reducing the therapeutic effect. It is used for the treatment of invasive fungal infections in patients who are refractory or intolerant to the conventional AmB therapy [23].

Amphotec^®^ is a formulation made up of AmB and cholesterol sulfate. It has a degree of antifungal efficacy similar to that of Fungizone^®^ but with less hemolytic and cytotoxic activity, particularly at the renal level, probably as a consequence of the strong affinity that exists between AmB and cholesterol, which decreases the amount of free AmB in the bloodstream [26]. Amphotec^®^ is used in the treatment of invasive aspergillosis in patients suffering renal disease or those who have low tolerability toward effective dosages of Fungizone^®^ and also in patients with aspergillosis resistant to Fungizone^®^ therapy.

Besides these formulations, liposomes have been used to improve the biopharmaceutical properties of AmB. These are self-assembled vesicles characterized by phospholipid bilayers containing aqueous environments and are suitable drug delivery systems (DDS) for many pharmaceutical applications [27,28,29,30,31]. In fact, their characteristics of biocompatibility and biodegradability, besides their peculiar structure, which is able to entrap hydrophilic, lipophilic and amphiphilic compounds, allow the efficient delivery of drugs with different physico-chemical and pharmacological properties, improving the drugs’ biopharmaceutical features and decreasing their side effects [32,33,34,35]. In particular, liposomes have been used as useful drug carriers in antitumor, antifungal, antimicrobial and antiviral applications [36,37]. For example, both Doxil^®^ (Janssen Products, Titusville, NJ, USA) and Caelyx^®^ (Schering-Plough, Kenilworth, NJ, USA), pharmaceuticals containing polyethylene-glycol-coated liposomes with mean sizes of ~100 nm, have been approved by the US Food and Drug Administration (F.D.A, Silver Spring, MD, USA) and the European Medicines Agency (E.M.A., London, UK) for the treatment of Kaposi Sarcoma and other cancerous diseases [38].

Ambisome^®^ is a liposomal formulation of AmB in which the drug is strongly associated with the bilayer structure of small unilamellar liposomes [39]. Ambisome^®^ has been marketed in Europe since 1989, and was approved in 1997 by the US F.D.A. for the treatment of visceral leishmaniasis. The formulation is made up of hydrogenated soy phosphatidylcholine, distearoylphosphatidylglycerol and cholesterol. It also contains other excipients such as antioxidants, α-tocopherol, disodium succinate hydrate and sucrose, as isotonic agents. Ambisome^®^ is characterized by a prolonged circulation time in the blood stream and is less toxic than Fungizone^®^. In a recent meta-analysis investigation, the renal toxicity and efficacy of Ambisome^®^ vs. free AmB^®^ were evaluated, evidencing a significant decrease in side effects for the liposomal formulation [40].

### 1.2. Nanotechnology in the Pharmaceutical Field

Nanotechnology is defined as the manipulation of matter on the atomic or molecular level while nanomedicine is its application in the prevention, diagnosis and treatment of diseases [41].

Current pharmacological therapy has provided evidence of many problems related to body distribution and stability of drugs in the blood stream. A strategic approach in the solution of these problems is based on the improvement of the selectivity and specificity of active compounds through the use of advanced drug delivery systems (DDS) [42]. The main advantages of using DDSs include (i) the opportunity of concentrating the encapsulated/complexed drug(s) in specific tissues through passive or active targeting; (ii) the modulation of the drug-release profile; and (iii) the protection of active compounds from the physiological metabolic processes with a resulting increase in the drug’s half-life. The polymeric nanoparticles have already been employed as drug delivery systems with great success, as a consequence of their advantages with respect to lipid formulations [41,42,43]. Nanoparticulate drug delivery systems have potential applications in many fields, including antitumor- and antiviral-therapies, genetic therapy, radiotherapy and treatment of CNS-diseases [42,44,45,46]. The vaccination and the immunomodulation promoted by nanoparticles are other emerging and important applications [47,48,49]. Nanoparticles possess unique features able to improve the administration of hydrophilic/lipophilic compounds and to allow selective targeting, after the conjugation of nanocarriers with specific targeting moieties on their surfaces [50,51].

## 2. Polymeric Nanoparticles

Polymeric nanoparticles are solid colloidal nanodevices with an average size ranging from 100 to 500 nm and are made up of different biomaterials [44,45,46,47,48,49,50,51,52]. The choice of which polymeric material to use depends on several different factors, namely (i) the compatibility of the proposed material with the compound(s) to be delivered; (ii) the required morphology of the nanosystems (capsules or matrixes) as a function of the release profile of the entrapped drug to be achieved (zero- or first-order, respectively) and (iii) the possibility of modifying the surfaces of the nanoparticles through chemical approaches or through physical interaction with specific molecules [46,50,51,52,53,54,55]. Many biocompatible polymers can be used to prepare nanoparticles and some polymer-based nanomedicines are already in the advanced clinical phase [56].

### 2.1. PLGA Nanoparticles Containing Amphotericin B

Among the synthetic polymers generally used to prepare nanoparticle-based drug delivery systems, poly(D,L-lactide) (PLA), poly(D,L-glycolide) (PLG) and their co-polymers poly(lactide-co-glycolide) (PLGA) are the most exploited because they are considered safe and they have been approved by the US Food and Drug Administration (FDA) as well as by the European regulatory authorities (EMA) for pharmaceutical application [57]. PLGA is normally metabolized into water and carbon dioxide by the body’s tricarboxylic acid cycle, sidestepping any possible accumulation phenomena and related side effects and is therefore used in drug delivery as an ideally safe material [58,59]. Therefore, the endocytic mechanism for the internalization of PLGA nanoparticles in cells occurs through several pathways such as fluid phase pinocytosis or clathrin-mediated processes, as a function of cell type and particle size. Following their uptake, the PLGA nanoparticles are localized in the endo-lysosomes, from which they escape into the cytosol within 10 min, due to the strong interaction between the nanoparticles and the vesicular membranes [53].

Polymeric nanoparticles were tested as nanocarriers for the delivery of antileishmanial compounds because of their ability to be internalized into infected cells, which favors the pharmacological effect of the entrapped compound [60].

The aim of this review is to provide a comprehensive description of the foremost recent investigations based on the encapsulation of anti-*Leishmania* compounds in PLGA nanoparticles in order to furnish an overview of the technological properties of novel colloidal formulations useful in the treatment of Leishmaniasis (Table 2). In particular, the approaches used to realize polyester-based nanoparticles containing amphotericin B will be examined and discussed.

In 2009, Amaral and coworkers described the preparation of polymeric blends (Nano-D-AmB) made up of PLGA and dimercapto-succinic acid (DMSA) containing Fungizone^®^ (desoxycholate AmB, D-AmB) with the aim of reducing the number of AmB administrations required in the treatment of paracoccidioidomycosis (PCM) [61]. The therapeutic efficacy and toxicity of this formulation (Nano-D-AmB) were compared with those of Fungizone^®^ (the conventional D-AmB) in a murine model of systemic PCM. The PLGA–DMSA polymeric nanoparticles loaded with D-AmB were prepared using the water-in-oil emulsification process, and their pharmacological efficacy was investigated on BALB/c mice infected with the Paracoccidioides brasiliensis yeast, in order to mime the chronic form of paracoccidioidomycosis. Specifically, 30 days post-infection, Nano-D-AmB (6 mg/kg every three days) was injected i.p. and the antifungal effect was compared to that of D-AmB (2 mg/kg a day). The results provided evidence of a comparable antifungal effect between the two formulations with, however, a decrease in the loss of body weight and absence of signs of stress (piloerection and hypotrichosis) in the case of Nano-D-AmB. No renal (blood urea nitrogen (BUN), creatinine) or hepatic (pyruvic and oxalacetic glutamic transaminases) biochemical abnormalities were observed for Nano-D-AmB either. The micronucleus assay showed no significant differences in either the micronucleus frequency or the percentage of polychromatic erythrocytes when Nano-D-AMB was used, thus providing evidence of the absence of both genotoxicity and cytotoxic effects. These results corroborated the efficacy of Nano-D-AmB as a novel antifungal nanomedicine, which significantly decreased both the AmB side effects and the number of administrations necessary [61].

Polymeric nanoparticles have also been proposed as useful devices for the oral administration of water-insoluble molecules with the aim of improving their bioavailability and decreasing their toxicity [62]. The ability of polymeric colloids to cross the mucosal barrier causes an increase in the systemic drug concentration as a consequence of distinctive uptake mechanisms [63,64]. The binding, uptake and absorption properties of polyester micro- and nanoparticles in CaCo-2 monolayers and were investigated in ileal tissue and gut-associated lymphoid tissue (GALT) of anaesthetized rats and rabbits, and it was demonstrated that (i) nanoparticles were absorbed better than microparticles and (ii) the uptake of PLA particles is obtained through the trans-cellular route. In particular, Italia and coworkers (2009) developed AmB-nanoparticles able to improve the drug’s oral bioavailability and minimize its side effects [65]. This polymeric nanomedicine was made up of AmB and PLGA and was prepared by emulsion–diffusion–evaporation and nanoprecipitation methods using Vitamin E-TPGS (D-α-Tocopherol polyethylene glycol 1000 succinate) as a stabilizer. The characterization of the nanosystems was carried out as a function of the differing amounts of AmB initially added (5, 10 and 15% *w*/*w* with respect to the polymer).

It was demonstrated that the preparation procedure influences the physico-chemical features of the formulations as well as the viscosity of the organic phase, which was shown to modify the colloidal mean sizes [66]. In particular, when 10% *w*/*w* of AmB was added, 1 mL of dimethyl sulfoxide (viscous in nature) in combination with ethyl acetate is required to solubilize the drug if the emulsion–diffusion–evaporation method is applied, while the nanoprecipitation method requires only 0.65 mL of DMSO in combination with acetone [65]. During experimentation, greater amounts of DMSO in the organic phase led to an increase in the organic phase viscosity, thus resulting in a significantly (*p* < 0.05) bigger particle diameter, 182.6 ± 6.0 nm with respect to 165.6 ± 2.8 (*p* < 0.05). Moreover, nanoparticles prepared with 10% and 15% *w*/*w* of AmB showed a significantly greater entrapment efficiency than nanosystems prepared with 5% *w*/*w* of AmB. Nanoparticles prepared with 15% *w*/*w* of AmB also showed higher PI values than the other formulations, probably as a consequence of the destabilization phenomena exerted by the hydrophobic drug on the particle structure. Atomic force microscopy (AFM) showed that these nanosystems have a smooth, spherical shape. The in vitro release of AmB showed that a biphasic drug leakage occurred, which was characterized by a rapid initial release of AmB followed by a sustained release. It was also interesting to note that the nanoparticles containing AmB elicited less hemolysis and nephrotoxicity than the Fungizone^®^ in Sprague–Dawley rats, while the relative drug bioavailability was found to be ~800% more than Fungizone^®^ following oral administration [65].

A follow-up study carried out by the same research team highlights ulterior optimization of the AmB formulation and its efficacy in murine models of disseminated and invasive pulmonary aspergillosis after oral administration [67]. In particular, the effects of different types of solvents (water, 50% *v*/*v* ethanol and pure ethanol) on particle size, size distribution and AMB entrapment efficiency were investigated. The results showed that the particle diameter decreased as follows: water ≥ 50% *v*/*v* ethanol ≥pure ethanol. The size distribution varied considerably as a function of the solvent, namely, pure ethanol resulted in a narrow distribution profile. No significant effect was observed on the entrapment efficiency (~70%) because the amount of DMSO remained constant. The study provided evidence of the crucial role the surfactant VE-TPGS plays in the formulation, which it does by stabilizing the AmB suspension, thus avoiding the appearance of aggregates.

In the case of an organic phase made up of a mixture of DMSO/acetone, the particle size and the entrapment efficiency decreased (from 160 ± 23 to 93 ± 14 nm and from 76 ± 11 to 64 ± 7%, respectively) when the amount of DMSO in the solvent mixture increased. This finding could have been due to the physico-chemical properties of the solvents and non-solvents used in the preparation of the nanoparticles, including the polarity and tonicity, both of which are influenced by the values of the basicity and acidity of the solvent. Namely, the decrease in the entrapment efficiency of AmB as a function of the increase in the DMSO fraction was probably due to the very good degree of solubility of AmB in the DMSO as compared to that in acetone. The effect of the solvent volume was also evaluated, that is, four volumes of DMSO (1, 2, 3 and 4 mL) were used to prepare the nanosystems containing AmB. The particle diameter (from 116 ± 22 to 86 ± 14 nm) and the entrapment efficiency (from 71 ± 9 to 54 ± 7%) decreased as the volume of the DMSO moved from 1 up to 4 mL. Moreover, the mean particle size proportionally increased (from 77 ± 10 to 113 ± 15 nm) with respect to the initial drug concentration used (10, 20, 30 and 40% *w*/*w* as a function of polymer weight) as did the entrapment efficiency which resulted in higher values (from 61 ± 6 to 71 ± 9).The formulation realized using 30% *w*/*w* of AmB was used in the in vivo investigations, evaluating its therapeutic efficacy in neutropenic murine models of disseminated and invasive pulmonary aspergillosis following oral administration. This formulation exhibited comparable or superior efficacy with respect to that of parenterally administered Ambisome^®^ or Fungizone^TM^, thus demonstrating the potential application of these nanosystems as useful nanocarriers for the oral delivery of AMB [67].

Moreover, PLGA nanoparticles and nanosuspensions containing AmB were also used as a valuable, cost-effective alternatives of Fungizone^TM^ and AmBisome^®^ by Van de Ven and coworkers [43]. They were prepared by means of the nanoprecipitation method, using PVA as stabilizer (0.5% *w*/*v*, MW 30–70 kDa) or surfactant mixtures of poloxamer 188 (P188), poloxamer 338 (P338) with polysorbate 80 (P80, Tween 80) or sodium cholate. The obtained nanoparticles were characterized by a mean diameter of 86–153 nm with a relatively narrow size distribution which was dependent on the type of stabilizer used: the smallest nanosystems were obtained using P188 in association with sodium cholate, whereas the largest ones were obtained with PVA. Nanoparticle formulations containing only P188 aggregated instantaneously. In this case, the addition of P80 or sodium cholate favored a decrease of the PI values to less than 0.10. PLGA nanoparticles stabilized with poloxamers, except in the case of P338/sodium cholate, were more negatively charged than nanoparticles stabilized with PVA. Namely, the P188/P80 mixtureprovides a zeta potential value of −31.4 ± 1.0 mV, which can be considered useful in assuring physical stability due to electrostatic repulsion, even though the nanoparticles aggregated upon purification. Contrarily, the PVA-stabilized nanoparticles (with a zeta potential of ~−9 mV), did not show any aggregation during cross-flow filtration and provided evidence of a high degree of stability upon storage at 4 °C for at least two weeks. The AmB-loaded PLGA nanoparticles prepared with a DMSO/acetone (1:1) mixture were larger than those prepared with DMSO alone.

In vitro sensibility tests were performed on MRC-5 cells, red blood cells, *Leishmania* infantum promastigotes and intracellular amastigotes, besides the fungal species Candida albicans, Aspergillus fumigatus and Trichophyton rubrum. The in vivo efficacy was assessed and compared to that of Fungizone and AmBisome in the acute *A. fumigatus* mouse model at a drug concentration of 2.5 and 5.0 mg/kg. The AmB-loaded PLGA nanoparticles were found to be about two times more efficacious in reducing the total burden as compared to AmBisome and Fungizone, therefore representing a potent and cost-effective alternative to the commercial formulations [43].

De Carvalho and coworkers designed a new nanoparticle formulation containing desoxycholate amphotericin B (D-AmB) with the aim of obtaining a successful release of the active agents and suitable antiprotozoal activity [68]. For this purpose, AmB nanoencapsulated in PLGA/dimercaptosuccinic acid (DMSA) nanoparticles (Nano-D-AmB) was developed, and its efficacy was evaluated in the treatment of experimental cutaneous leishmaniasis in C57BL/6 mice. This was to investigate whether the nanosystems could decrease the administration regimen while maintaining the same therapeutic effects as the free D-AmB. Magnetic citrate-coated maghemite nanoparticles were added to this nanosystem (Nano-D-AmB-MG), in an attempt to increase the controlled release of AmB through magnetohyperthermia. The Nano-D-AmB was prepared according to the procedure already described by Amaral and coworkers with a slight modification [61]. TEM and SEM analyses provided evidence of a narrow size distribution of the nanoparticles and demonstrated that the presence of magnetic nanoparticles induced an increase in the mean diameter as a consequence of particle aggregation related to the effect of the magnetic fluid (particle growth) [68].

**Figure 2:**
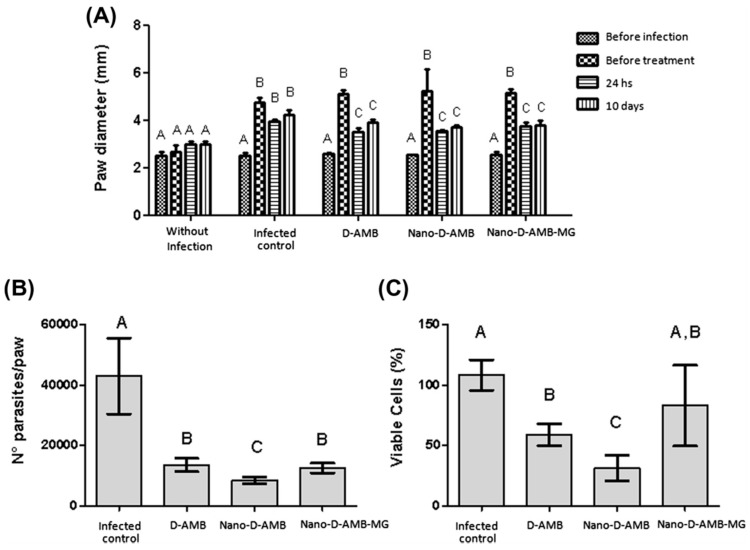
Distribution of the paw diameter (mm) of the mice according to duration of treatment (**A**), number of parasites per paw (**B**), and percentage of viable cells (**C**). Without infection = negative control group; *Leishmania* = infected animals treated with% PBS; D-AMB = infected animals treated with free D-AMB; Nano-D-AMB = infected animals treated with Nano-D-AMB; Nano-D-AMB-MG = infected animals treated with Nano-D-AMB-MG and AC magnetic field to produce magnetohyperthermia. Different letters indicate significant differences (*p* < 0.05) detected by the Tukey post hoc test, where comparisons were made between the treatment groups (**A**,**B**) and also within the groups: before infection, before treatment, 24 h and 10 days (**A**). Bar graphs were expressed as standard deviation (from [68]).

Female C57BL/6 mice were infected intradermally in the right footpad with promastigotes of *Leishmania* amazonensis in the metacyclic phase. The infected animals were divided into four groups to be treated intraperitoneally, as reported in Figure 2. The first group with 1% PBS for ten consecutive days; the second group with D-AMB at 2 mg/kg/day for 10 days (totalizing 20 mg/kg/animal); the third group with Nano-D-AMB and the fourth group with Nano-D-AMB-MG (3.4 × 10^13^ particles/mL) at 6 mg/kg on the 1st, 4th and 7th days and at 2 mg/kg on the 10th day, totalizing 20 mg/kg/animal by the end of the treatment. The Nano-D-AMB-MG group was submitted to an alternating current (AC) magnetic field (40 Oe amplitude AC magnetic field oscillating at 1 MHz) in order to generate the necessary magnetohyperthermia. The results were expressed as a function of paw diameter measurements, parasite number and cell viability. The D-AMB-coated PLGA–DMSA nanoparticles showed the same efficacy as the free D-AMB in reducing paw diameter. In addition, the Nano-D-AMB treatment elicited a greater reduction in the number of parasites and cell viability with respect to the free D-AMB. This result suggests that the nanoparticles were more efficacious than the free D-AMB, allowing the reduction of the frequency of administration [68].

The magnetic nanoparticles did not show the expected results, probably as a consequence of the observed aggregation effect, which could have been responsible for the decreased degree of target delivery and hence for the reduced drug efficacy. Moreover, the mice were exposed for only 10 min to the AC magnetic field in order to produce the desired magnetohyperthermia, and perhaps this was not enough to allow the intraperitoneally-administered magnetic nanoparticles to reach the lesion under suitable conditions [69].

### 2.2. PLGA-PEG Nanoparticles Containing Amphotericin B

Several efforts have been made in order to improve the bioavailability of amphotericin B by avoiding the rapid uptake by phagocytic cells using poly(ethylene glycol) (PEG). In particular, Al-Quadeib et al*.* described a new oral AmB-nanoformulation made up of PLGA-PEG 15%-diblock copolymer using a modified emulsification method [70]. The high percentages of PEG residues of copolymer together with the elevated drug content provided a significant decrease of MPS uptake (70%) with respect to other nanoparticle formulations containing AmB [70]. The spherical particles of AmB varied in size from 26.4 ± 2.9 to 1068 ± 489.8 nm. The formulation containing three surfactants in the aqueous phase, in particular PVP (polyvinyl pirrolidone), TPGS (vitamin E) and Pluronic F68, favored a decrease in their mean sizes and an improvement in drug retention with respect to the formulation prepared with the PVP alone. Fourier Transform Infra-Red Spectroscopy (FTIR) showed the occurrence of an electrostatic interaction between the AmB and PLGA-PEG copolymer, which promoted a successful interaction between the drug and the polymer. Moreover, a biphasic release of the drug from the nanoparticles characterized by an initial burst release of the compound was observed, followed by a slow release phase over the next 24 h. These results clearly showed a controlled release of the drug by stabilized PLGA-PEG nanosystems, providing the rationale of a plausible oral administration of entrapped AmB for the treatment of fungal- and leishmanial-related diseases [70].

Recently, Carraro and coworkers performed an investigation concerning an experimental designable to assess the ideal formulation of PLGA and PLGA-PEG nanoparticles containing AmB through the emulsion–solvent–evaporation method, and evaluated the influence of several factors on the properties of the obtained nanosystems, such as the mean diameter and drug encapsulation efficiency [71,72]. The formulations prepared using PLGA showed an average size of ~200 nm, with a polydispersity index of 0.12 ± 0.02. Various parameters were modulated in order to obtain formulations that could be systemically administered (i.e., the kind of organic solvent used such as chloroform, ethyl acetate and methanol, or the concentration of the polymer used). The results showed that methanol increased the particle size of the PLGA nanoparticles, while ethyl acetate and chloroform favored a significant decrease in the mean diameter of the systems. Moreover, the average size of the PLGA-PEG nanoparticles containing AmB was ~160 nm with a low polydispersity index and the analyses showed that, again, the best solvents to be used for obtaining small nanosystems were ethyl acetate followed by chloroform [68]. The encapsulation efficiency was high in both the PLGA and PLGA-PEG-formulations; in particular, ethyl acetate and methanol provided the best values of drug retention when they were used as cosolvents, as opposed to chloroform. Considering the obtained data, the ideal PLGA-PEG formulation able to efficiently deliver AmB for the treatment of Leishamaniasis requires the use of ethyl acetate as cosolvent and no more than 50 mg of polymer during the sample preparation [71,72].

In addition, Kumar et al. recently demonstrated that the toxicity of AmB-loaded PLGA-PEG nanoparticles against extracellular promastigotes was lower compared to that of the free drug, while the inhibition of amastigote activity localized in the spleens of the animals was significantly greater [73].

### 2.3. Selective Targeting of PLGA-Nanoparticles Containing Amphotericin B

The fact that particulate carriers are taken up by the mononuclear phagocyte system (MPS) in the absence of a specific coating makes them ideal vehicles for selective drug delivery in tissues rich in phagocytic cells, such as the liver, spleen, and lungs. In fact, the nature of these systems allows the localization of the entrapped drug(s) inside the macrophages, which may harbor many of the important pathogens in their intracellular compartments, such as *leishmania* [74]. On the other hand, the targeting of specific receptors represents a consolidated strategy able to increase the concentration of colloids in a tissue [31,75].

**Figure 3:**
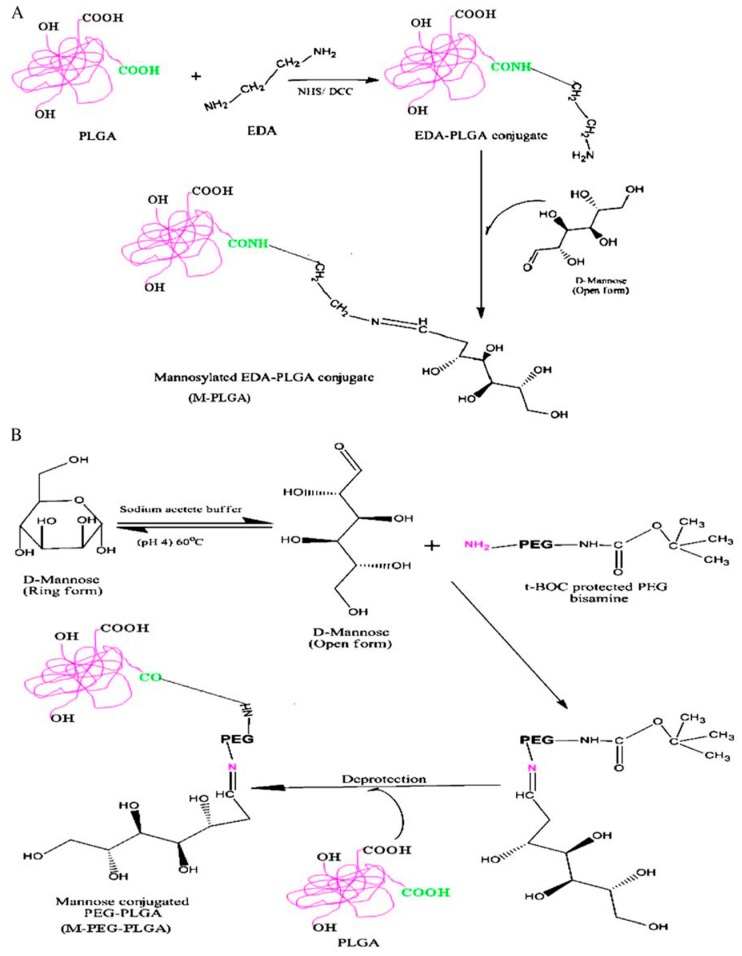
Scheme of synthesis of mannose–PLGA conjugates; (**A**) without spacer (M-PLGA); (**B**) with PEG spacer (M-PEG-PLGA) (from [76]).

The mannose receptors present on the macrophage membrane furnished the ideal place to begin for incrementing the localization of nanoparticulate delivery systems. In their studies, Nahar and Jain (2009) developed mannose-anchored PLGA nanoparticles, with the aim of increasing the delivery of amphotericin B to the macrophages, thus significantly enhancing the efficacy of the drug in the treatment of macrophage-specific diseases such as VL [76]. The influence of the spacer between the mannose and the particle surface on macrophage targeting was also evaluated (Figure 3). PLGA was linked to mannose by direct conjugation (M-PLGA) or through the use of a PEG spacer (MPEG-PLGA). The nanoparticles were prepared following the emulsion–solvent evaporation technique using sodium cholate as surfactant. The mean sizes of blank PLGA nanoparticles, M-PLGA nanoparticles and M-PEG PLGA nanosystems were found to be 146 ± 26, 157 ± 12.2 and 178 ± 10.4 nm, respectively. The diameter of the nanoparticles did not increase much after the encapsulation of AmB probably as a consequence of the monomeric form of the AmB during the formation of the nanoparticles (Table 2) [76]. The polydispersity index of the nanosystems was very low, while the M-PLGA and M-PEG-PLGA nanoparticles showed a marginal increase of this parameter. The zeta potential of PLGA-, M-PLGA-, and M-PEG-PLGA nanoparticles was found to be −43.04 ± 1.4, −26.9 ± 1.4, −34.9 ± 1.9 mV, respectively. The AmB induced a slight increase in the surface charge of all the formulations (Table 2). Transmission electron microscopy (TEM) and atomic force microscopy (AFM) showed that the M-PLGA- and M-PEG-PLGA nanoparticles were spherical in shape and in the nanometric range, while the surfaces of the engineered nanoparticles were rather less smooth with the occurrence of a slight degree of aggregation in comparison to the PLGA nanosystems. Then, macrophage targeting was evaluated via cellular uptake, ex vivo antileishmanial activity and in vivo biodisposition patterns of engineered nanoparticles in macrophage-rich organs. The results of the ex vivo antileishmanial activity showed a greater efficacy of plain and engineered AmB-loaded nanoparticles in an intra-amastigote macrophage model as compared to the free drug as follows: M-PEG-PLGA nanoparticles > M-PLGA nanoparticles and PLGA-nanoparticles [76]. The greater efficacy of the M-PEG-PLGA nanoparticles is related to its more efficient cellular uptake in comparison to the other formulations, thus confirming that the spacer provides flexibility, accessibility and minimal steric hindrance to ligands, all of which allow it to efficiently interact with the receptor [77,78]. The biodistribution data, obtained by inoculating Swiss albino mice with the formulations, showed that mannosylated nanoparticles with spacers possess the best targeting features, made evident by the quantification of the highest concentration of administered dosages in macrophage-rich tissues. This approach has been used by other research teams in order to efficiently deliver AmB by means of polymeric systems. For example, Shahnaz et al. demonstrated that the mannosylation of thiolated chitosan (TC) nanoparticles (MTC) could be a useful approach for improving AmB-intramacrophage localization and the treatment of Visceral Leishmaniasis (VL) [79]. A sustained drug release for up to 10 days was observed from TC and MTC nanocarriers with respect to unmodified chitosan nanocarriers (UC), which carried out sustained drug release for only four days. This phenomenon was probably due to the covalent cross-linking of disulfide bonds in the modified polymer matrix during the swelling process. Moreover, the MTC formulation showed the best biocompatibility on J774 (macrophagic cell lines) with respect to TC systems and unmodified particles, and better pharmacological activity with respect to AmBisome, confirming the potentiality of mannosylation as a useful approach for the treatment of intracellular infections [79].

## 3. Conclusions

Pharmaceutical nanotechnology is emerging more and more as a novel approach in the improvement of the biopharmaceutical properties of drugs. Intracellular infections represent a challenging issue to be solved as a consequence of the poor cytosolic localization of many active compounds. The application of nanoparticles acting as “Trojan horses” in the veterinary sector could allow the administration of active compounds characterized by different physico-chemical properties, favor their cellular uptake, allow the selective targeting of specific body compartments of animals by the functionalization of their surfaces, decrease the administration regimen and, finally, decrease costs. Even though much progress has been made through the use of innovative nanocarriers, the development of novel and more efficacious nanomedicines is a fundamental requirement because knowledge of the bio-mechanisms of many intracellular parasites remains obscure. Unfortunately, no type of systemic administration of PLGA nanoparticles has been approved for clinical use up to now, probably because there have been diverse sticking points in clinical translation with regards to both delivery aspects (e.g., biological challenges) and regulatory aspects (e.g., study design and approval challenges). In fact, there is a general lack of regulatory standards regarding the examination of nanoparticle-based therapeutics, so significant efforts are being made in this direction [80]. Moreover, the development of polymeric nanosystems containing specific markers or compounds could provide opportunities for managing novel nanotheranostic formulations able to detect and treat many types of diseases [81].

## Figures and Tables

**Table 1:** Main features of AmB formulations.

Name	Formulation	Composition	Application	Ref.
**Fungizone^®^**	Colloidal dispersion	AmB, sodium deoxycholate (1:2 molar ratio)	Treatment of invasive fungal infections; treatment of leishmaniasis (not as primary therapy)	[23,24,25]
**Ambisome^®^**	Unilamellar liposomes	Phosphatidylcholine, cholesterol, diastearoylglycerol and AmB (2:1:0.8:0.4 molar ratio)	Therapy of febrile neutropenia, aspergillosis, candidiasis, and cryptococcosis; treatment of visceral leishmaniasis (as second-line therapy)	[39]
**Albecet^®^**	Lipid complex	AmB, L-α dimyristoylphosphatidylcholine, L-α dimyristoylphosphatidylglycerol (1:7:3 molar ratio)	Treatment of invasive fungal infections in patients resistant or intolerant to conventional AmB therapy	[23]
**Amphotec^®^**	Colloidal lipid complex	AmB, cholesterol sulfate (1:1 molar ratio)	Similar antifungal efficacy of Fungizone^®^ but less hemolytic and cytotoxic effects.	[26]

**Table 2:** Main features of AmB-Nanoparticles.

Composition	Preparation	Size (nm)	ZP (mV) ^a^	Administration Route	Ref.
AmB, PLGA, PVA	Nanoprecipitation method	86–153	−9	i.p.	[43]
AmB, PLGA, P188-P338 with Tween80 or Sodium cholate	Nanoprecipitation method	86–153	−31	i.p.	[43]
D-AmB ^b^, PLGA, DMSA ^c^	Water-in-oil emulsification and solvent evaporation method	--	-	i.p.	[61]
AmB, PLGA, Vitamin E-TPGS	Emulsion-diffusion evaporation method Nanoprecipitation method	182 165	−16 −15	Oral	[65]
AMB, PLGA, Vitamin E-TPGS, DMSO, Ethanol, AmB	Emulsion-diffusion evaporation method	113	-	Oral	[65]
AmB, PLGA, Sodium cholate	Emulsion solvent evaporation method (o/w emulsification)	154	−46	i.v.	[67]
AmB, diblock polymer PLGA–PEG with 15% PEG	Modified emulsification method	26 to 1068	-	Oral	[70]
Mannosylated	Emulsion solvent evaporation method	157	−26	i.v.	[76]
PEG-Mannosylated	Emulsion solvent evaporation method	178	−34	i.v.	[76]
Mannose-anchored thiolated chitosan	Emulsion solvent evaporation method	362	-	i.v.	[79]

^a^ Zeta potential; ^b^ desoxycholate AmB; ^c^ dimercapto-succinic acid.

## References

[1] Bern C., Maguire J.H., Alvar J. (2008). Complexities of assessing the disease burden attributable to leishmaniasis. PLoS Negl. Trop. Dis..

[2] Alvar J., Vélez I.D., Bern C., Herrero M., Desjeux P., Cano J., Jannin J., den Boer M., WHO Leishmaniasis Control Team (2012). Leishmaniasis worldwide and global estimates of its incidence. PLoS ONE.

[3] World Health Organization Leishmaniasis. http://apps.who.int/tdr/svc/diseases/leishmaniasis/.

[4] Singh N., Kumar M., Singh R.K. (2012). Leishmaniasis: Current status of available drugs and new potential drug targets. Asian Pac. J. Trop. Med..

[5] Lipoldová M., Demant P. (2006). Genetic susceptibility to infectious disease: Lessons from mouse models of leishmaniasis. Nat. Rev. Genet..

[6] Croft S.L., Sundar S., Fairlamb A.H. (2006). Drug resistance in leishmaniasis. Clin. Microbiol. Rev..

[7] Frézard F., Demicheli C., Ribeiro R.R. (2009). Pentavalent antimonials: New perspectives for old drugs. Molecules.

[8] Guerin P.J., Olliaro P., Sundar S., Boelaert M., Croft S.L., Desjeux P., Wasunna M.K., Bryceson A.D. (2002). Visceral leishmaniasis: Current status of control, diagnosis, and treatment, and a proposed research and development agenda. Lancet Infect. Dis..

[9] Maltezou H.C. (2010). Drug resistance in visceral leishmaniasis. J. Biomed. Biotechnol..

[10] Sundar S., More D.K., Singh M.K., Singh V.P., Sharma S., Makharia A., Kumar P.C., Murray H.W. (2000). Failure of pentavalent antimony in visceral leishmaniasis in India: Report from the center of the Indian epidemic. Clin. Infect. Dis..

[11] Miró G., Cardoso L., Pennisi M.G., Oliva G., Baneth G. (2008). Canine leishmaniosis-new concepts and insights on an expanding zoonosis: Part two. Trends Parasitol..

[12] Duffin J., Renè P. (1991). Anti-moine; anti-biotique: The public fortunes of the secret properties of antimony potassium tartrate (tartar emetic). J. Hist. Med. Allied Sci..

[13] Bern C., Adler-Moore J., Berenguer J., Boelaert M., den Boer M., Davidson R.N., Figueras C., Gradoni L., Kafetzis D.A., Ritmeijer K. (2006). Liposomal amphotericin B for the treatment of visceral leishmaniasis. Clin. Infect. Dis..

[14] Laniado-Laborín R., Cabrales-Vargas M.N. (2009). Amphotericin B: Side effects and toxicity. Rev. Iberoam. Micol..

[15] Davis A.J., Kedzierski L. (2005). Recent advances in antileishmanial drug development. Curr. Opin. Investig. Drugs..

[16] Sundar S., Jha T.K., Thakur C.P., Bhattacharya S.K., Rai M. (2006). Oral miltefosine for the treatment of Indian visceral leishmaniasis. Trans. R. Soc. Trop. Med. Hyg..

[17] Sundar S., Murray H.W. (2005). Availability of miltefosine for the treatment of kala-azar in India. Bull. World Health Organ..

[18] Oliva G., Roura X., Crotti A., Maroli M., Castagnaro M., Gradoni L., Lubas G., Paltrinieri S., Zatelli A., Zini E. (2010). Guidelines for treatment of leishmaniasis in dogs. J. Am. Vet. Med. Assoc..

[19] Berczi I., Bertók L., Chow D.A., Chow A. (2000). Natural immunity and neuroimmune host defense. N. Y. Acad. Sci..

[20] Gómez-Ochoa P., Castillo J.A., Gascón M., Zarate J.J., Alvarez F., Couto C.G. (2009). Use of domperidone in the treatment of canine visceral leishmaniasis: A clinical trial. Vet. J..

[21] Baneth G., Shaw S.E. (2002). Chemotherapy of canine leishmaniosis. Vet. Parasitol..

[22] Paila Y.D., Saha B., Chattopadhyay A. (2010). Amphotericin B inhibits entry of Leishmania donovani into primary macrophages. Biochem. Biophys. Res. Commun..

[23] Torrado J.J., Espada R., Ballesteros M.P., Torrado-Santiago S. (2008). Amphotericin B formulations and drug targeting. J. Pharm. Sci..

[24] Lemke A., Kiderlen A.F., Kayser O. (2005). Amphotericin B. Appl. Microbiol. Biotechnol..

[25] Caillot D., Chavanet P., Casasnovas O., Solary E., Zanetta G., Buisson M., Wagner O., Cuisenier B., Bonnin A., Camerlynck P. (1992). Clinical evaluation of a new lipid-based delivery system for intravenous administration of amphotericin B. Eur. J. Clin. Microbiol. Infect. Dis..

[26] Veerareddy P.R., Vobalaboina V. (2004). Lipid-based formulations of amphotericin B. Drugs Today.

[27] Askarizadeh A., Jaafari M.R., Khamesipour A., Badiee A. (2017). Liposomal adjuvant development for leishmaniasis vaccines. Ther. Adv. Vaccines.

[28] Simões S., Filipe A., Faneca H., Mano M., Penacho N., Düzgünes N., de Lima M.P. (2005). Cationic liposomes for gene delivery. Expert Opin. Drug Deliv..

[29] Vyas K.S., Rajendran S., Morrison S.D., Shakir A., Mardini S., Lemaine V., Nahabedian M.Y., Baker S.B., Rinker B.D., Vasconez H.C. (2016). Systematic review of liposomal bupivacaine (exparel) for postoperative analgesia. Plast. Reconstr. Surg..

[30] Cosco D., Tsapis N., Nascimento T.L., Fresta M., Chapron D., Taverna M., Arpicco S., Fattal E. (2017). Polysaccharide-coated liposomes by post-insertion of a hyaluronan-lipid conjugate. Colloids Surf. B Biointerfaces.

[31] Paolino D., Cosco D., Gaspari M., Celano M., Wolfram J., Voce P., Puxeddu E., Filetti S., Celia C., Ferrari M. (2014). Targeting the thyroid gland with thyroid-stimulating hormone (TSH)-nanoliposomes. Biomaterials.

[32] Celano M., Schenone S., Cosco D., Navarra M., Puxeddu E., Racanicchi L., Brullo C., Varano E., Alcaro S., Ferretti E. (2008). Cytotoxic effects of a novel pyrazolopyrimidine derivative entrapped in liposomes in anaplastic thyroid cancer cells in vitro and in xenograft tumors in vivo. Endocr. Relat. Cancer.

[33] Cosco D., Paolino D., Maiuolo J., Russo D., Fresta M. (2011). Liposomes as multicompartmental carriers for multidrug delivery in anticancer chemotherapy. Drug Deliv. Transl. Res..

[34] Cristiano M.C., Cosco D., Celia C., Tudose A., Mare R., Paolino D., Fresta M. (2017). Anticancer activity of all-trans retinoic acid-loaded liposomes on human thyroid carcinoma cells. Colloids Surf. B Biointerfaces.

[35] Cosco D., Paolino D., Maiuolo J., Marzio L.D., Carafa M., Ventura C.A., Fresta M. (2015). Ultradeformable liposomes as multidrug carrier of resveratrol and 5-fluorouracil for their topical delivery. Int. J. Pharm..

[36] Celia C., Cosco D., Paolino D., Fresta M. (2011). Gemcitabine-loaded innovative nanocarriers vs. GEMZAR: Biodistribution, pharmacokinetic features and in vivo antitumor activity. Expert Opin. Drug Deliv..

[37] Paolino D., Cosco D., Molinaro R., Celia C., Fresta M. (2011). Supramolecular devices to improve the treatment of brain diseases. Drug Discov. Today.

[38] Barenholz Y. (2012). Doxil^®^—The first FDA-approved nano-drug: Lessons learned. J. Control. Release.

[39] Adler-Moore J., Proffitt R.T. (2002). AmBisome: Liposomal formulation, structure, mechanism of action and pre-clinical experience. J. Antimicrob. Chemother..

[40] Steimbach L.M., Tonin F.S., Virtuoso S., Borba H.H., Sanches A.C., Wiens A., Fernandez-Llimós F., Pontarolo R. (2017). Efficacy and safety of amphotericin B lipid-based formulations-A systematic review and meta-analysis. Mycoses.

[41] Hullmann A. (2006). Who is winning the global nanorace?. Nat. Nanotechnol..

[42] Tiwari G., Tiwari R., Sriwastawa B., Bhati L., Pandey S., Pandey P., Bannerjee S.K. (2012). Drug delivery systems: An updated review. Int. J. Pharm. Investig..

[43] Van de Ven H., Paulussen C., Feijens P.B., Matheeussen A., Rombaut P., Kayaert P., Van den Mooter G., Weyenberg W., Cos P., Maes L. (2012). PLGA nanoparticles and nanosuspensions with amphotericin B: Potent in vitro and in vivo alternatives to Fungizone and AmBisome. J. Control. Release.

[44] Cosco D., Federico C., Maiuolo J., Bulotta S., Molinaro R., Paolino D., Tassone P., Fresta M. (2014). Physicochemical features and transfection properties of chitosan/poloxamer 188/poly(D, L-lactide-co-glycolide) nanoplexes. Int. J. Nanomed..

[45] Paolino D., Cosco D., Celano M., Moretti S., Puxeddu E., Russo D., Fresta M. (2013). Gemcitabine-loaded biocompatible nanocapsules for the effective treatment of human cancer. Nanomedicine.

[46] Cosco D., Di Marzio L., Marianecci C., Trapasso E., Paolino D., Celia C., Carafa M., Fresta M. (2014). Colloidal supramolecular aggregates for therapeutic application in neuromedicine. Curr. Med. Chem..

[47] Silva J.M., Videira M., Gaspar R., Préat V., Florindo H.F. (2013). Immune system targeting by biodegradable nanoparticles for cancer vaccines. J. Control. Release.

[48] Sarti F., Perera G., Hintzen F., Kotti K., Karageorgiou V., Kammona O., Kiparissides C., Bernkop-Schnürch A. (2011). In vivo evidence of oral vaccination with PLGA nanoparticles containing the immunostimulant monophosphoryl lipid A. Biomaterials.

[49] Rietscher R., Schröder M., Janke J., Czaplewska J., Gottschaldt M., Scherließ R., Hanefeld A., Schubert U.S., Schneider M., Knolle P.A. (2016). Antigen delivery via hydrophilic PEG-b-PAGE-b-PLGA nanoparticles boosts vaccination induced T cell immunity. Eur. J. Pharm. Biopharm..

[50] Abed N., Couvreur P. (2014). Nanocarriers for antibiotics: A promising solution to treat intracellular bacterial infections. Int. J. Antimicrob. Agents.

[51] Gu W., Wu C., Chen J., Xiao Y. (2013). Nanotechnology in the targeted drug delivery for bone diseases and bone regeneration. Int. J. Nanomed..

[52] Grottkau B.E., Cai X., Wang J., Yang X., Lin Y. (2013). Polymeric nanoparticles for a drug delivery system. Curr. Drug Metab..

[53] Danhier F., Ansorena E., Silva J.M., Coco R., Le Breton A., Préat V. (2012). PLGA-based nanoparticles: An overview of biomedical applications. J. Control. Release.

[54] Diou O., Tsapis N., Giraudeau C., Valette J., Gueutin C., Bourasset F., Zanna S., Vauthier C., Fattal E. (2012). Long-circulating perfluorooctyl bromide nanocapsules for tumor imaging by 19FMRI. Biomaterials.

[55] Cosco D., Paolino D., De Angelis F., Cilurzo F., Celia C., Di Marzio L., Russo D., Tsapis N., Fattal E., Fresta M. (2015). Aqueous-core PEG-coated PLA nanocapsules for an efficient entrapment of water soluble anticancer drugs and a smart therapeutic response. Eur. J. Pharm. Biopharm..

[56] Svenson S. (2012). Clinical translation of nanomedicines. Curr. Opin. Solid State Mat. Sci..

[57] Makadia H.K., Siegel S.J. (2011). Poly Lactic-co-Glycolic Acid (PLGA) as biodegradable controlled drug delivery carrier. Polymers.

[58] Shive M.S., Anderson J.M. (1997). Biodegradation and biocompatibility of PLA and PLGA microspheres. Adv. Drug Deliv. Rev..

[59] Cosco D., Cilurzo F., Maiuolo J., Federico C., Di Martino M.T., Cristiano M.C., Tassone P., Fresta M., Paolino D. (2015). Delivery of miR-34a by chitosan/PLGA nanoplexes for the anticancer treatment of multiple myeloma. Sci. Rep..

[60] Costa Lima S., Rodrigues V., Garrido J., Borges F., Kong Thoo Lin P., Cordeiro da Silva A. (2012). In vitro evaluation of bisnaphthalimidopropyl derivatives loaded into pegylated nanoparticles against Leishmania infantum protozoa. Int. J. Antimicrob. Agents.

[61] Amaral A.C., Bocca A.L., Ribeiro A.M., Nunes J., Peixoto D.L., Simioni A.R., Primo F.L., Lacava Z.G., Bentes R., Titze-de-Almeida R. (2009). Amphotericin B in poly(lactic-co-glycolic acid) (PLGA) and dimercaptosuccinic acid (DMSA) nanoparticles against paracoccidioidomycosis. J. Antimicrob. Chemother..

[62] Kumar G., Shafiq N., Malhotra S. (2012). Drug-loaded PLGA nanoparticles for oral administration: Fundamental issues and challenges ahead. Crit. Rev. Ther. Drug Carr. Syst..

[63] McClean S., Prosser E., Meehan E., O’Malley D., Clarke N., Ramtoola Z., Brayden D. (1998). Binding and uptake of biodegradable poly-DL-lactide micro- and nanoparticles in intestinal epithelia. Eur. J. Pharm. Sci..

[64] Mittal G., Sahana D.K., Bhardwaj V., Ravi Kumar M.N. (2007). Estradiol loaded PLGA nanoparticles for oral administration: Effect of polymer molecular weight and copolymer composition on release behavior in vitro and in vivo. J. Control. Release.

[65] Italia J.L., Yahya M.M., Singh D., Ravi Kumar M.N. (2009). Biodegradable nanoparticles improve oral bioavailability of amphotericin B and show reduced nephrotoxicity compared to intravenous Fungizone. Pharm. Res..

[66] Sahana D.K., Mittal G., Bhardwaj V., Kumar M.N. (2008). PLGA nanoparticles for oral delivery of hydrophobic drugs: influence of organic solvent on nanoparticle formation and release behavior in vitro and in vivo using estradiol as a model drug. J. Pharm. Sci..

[67] Italia J.L., Sharp A., Carter K.C., Warn P., Kumar M.N. (2011). Peroral amphotericin B polymer nanoparticles lead to comparable or superior in vivo antifungal activity to that of intravenous Ambisome^®^ or Fungizone™. PLoS ONE.

[68] de Carvalho R.F., Ribeiro I.F., Miranda-Vilela A.L., de Souza Filho J., Martins O.P., Cintra e Silva Dde O., Tedesco A.C., Lacava Z.G., Báo S.N., Sampaio R.N. (2013). Leishmanicidal activity of amphotericin B encapsulated in PLGA–DMSA nanoparticles to treat cutaneous leishmaniasis in C57BL/6 mice. Exp. Parasitol..

[69] Hayashi K., Nakamura M., Miki H., Ozaki S., Abe M., Matsumoto T., Sakamoto W., Yogo T., Ishimura K. (2014). Magnetically responsive smart nanoparticles for cancer treatment with a combination of magnetic hyperthermia and remote-control drug release. Theranostics.

[70] Al-Quadeib B.T., Radwan M.A., Siller L., Horrocks B., Wright M.C. (2015). Stealth Amphotericin B nanoparticles for oral drug delivery: In vitro optimization. Saudi Pharm. J..

[71] Carraro T.C., Khalil N.M., Mainardes R.M. (2016). Amphotericin B-loaded polymeric nanoparticles: formulation optimization by factorial design. Pharm. Dev. Technol..

[72] Moraes Moreira Carraro T.C., Altmeyer C., Maissar Khalil N., Mara Mainardes R. (2017). Assessment of in vitro antifungal efficacy and in vivo toxicity of Amphotericin B-loaded PLGA and PLGA-PEG blend nanoparticles. J. Mycol. Med..

[73] Kumar R., Sahoo G.C., Pandey K., Das V., Das P. (2015). Study the effects of PLGA-PEG encapsulated amphotericin B nanoparticle drug delivery system against Leishmania donovani. Drug Deliv..

[74] Owais M., Gupta C.M. (2005). Targeted drug delivery to macrophages in parasitic infections. Curr. Drug Deliv..

[75] Alibakhshi A., Abarghooi Kahaki F., Ahangarzadeh S., Yaghoobi H., Yarian F., Arezumand R., Ranjbari J., Mokhtarzadeh A., de la Guardia M. (2017). Targeted cancer therapy through antibody fragments-decorated nanomedicines. J. Control. Release.

[76] Nahar M., Jain N.K. (2009). Preparation, characterization and evaluation of targeting potential of amphotericin B-loaded engineered PLGA nanoparticles. Pharm. Res..

[77] Sharma A., Sharma S., Khuller G.K. (2004). Lectin-functionalized poly (lactide-co-glycolide) nanoparticles as oral/aerosolized antitubercular drug carriers for treatment of tuberculosis. J. Antimicrob. Chemother..

[78] Kassab R., Parrot-Lopez H., Fessi H., Menaucourt J., Bonaly R., Coulon J. (2002). Molecular recognition by Kluyveromyces of amphotericin B-loaded, galactose-tagged, poly (lactic acid) microspheres. Bioorg. Med. Chem..

[79] Shahnaz G., Edagwa B.J., McMillan J., Akhtar S., Raza A., Qureshi N.A., Yasinzai M., Gendelman H.E. (2017). Development of mannose-anchored thiolated amphotericin B nanocarriers for treatment of visceral leishmaniasis. Nanomedicine.

[80] Anselmo A.C., Mitragotri S. (2016). Nanoparticles in the clinic. Bioeng. Transl. Med..

[81] Cosco D., Fattal E., Fresta M., Tsapis N. (2015). Perfluorocarbon-loaded micro and nanosystems for medical imaging: A state of the art. J. Fluor. Chem..

